# Hubs for Interactive Literature (HILs) as a Complimentary Visual Tool for Reviews

**DOI:** 10.3390/microplastics3020018

**Published:** 2024-05-29

**Authors:** Katie Stallings, Kimberly J. Zaccaria, Megan Riccardi, Gregory M. Zarus, Gaston Casillas

**Affiliations:** 1 Agency for Toxic Substances and Disease Registry (ATSDR), Office of Innovation and Analytics, Atlanta, GA 30341, USA; 2 Oak Ridge Institute for Science and Education (ORISE), Agency for Toxic Substances and Disease Registry (ATSDR), Atlanta, GA 30341, USA; 3 SRC Inc., North Syracuse, NY 13212, USA

**Keywords:** ATSDR, microplastics, HIL, review

## Abstract

In the last few years, microplastics research has exploded, with the field exploring new procedures and techniques that focus on a variety of scientific and policy issues. As there are not standardized definitions for many terms in the field, including the term microplastic itself, researchers utilize the same labels to describe different aspects of microplastic pollution. Here we provide a visual tool, called a Hub for Interactive Literature (HIL), to assist researchers in identifying and targeting specific literature. Currently, there are four Hubs for Interactive Learning (HILs) corresponding to previously published reviews, including a scoping review of microplastics literature as well as three reviews examining the human exposure and health effects of microplastics, the unique liver carcinogenicity of polyvinyl chloride (PVC) microplastics, and micro and nanoplastics found in the air. The HILs incorporate all of the literature used to produce the corresponding reviews. A couple of advantages that HILs provide in their capacity as a supportive instrument are the filtering options and easily accessed original references. This tool can be leveraged by researchers to rapidly review microplastics research and isolate specific subtopics of interest to develop new conclusions and quickly identify data gaps. We give an in-depth look at the HIL corresponding to a scoping review of microplastics literature to exhibit the novel functionality and advantages of this exciting tool. We demonstrate a novel world map of the literature to show that microplastics are a global scientific and public health issue. The map offers the additional functionality of filtering the references by country. We also provide a brief description of the current HILs to show the flexibility and personalization available when using this method.

## 1. Introduction

The Agency for Toxic Substances and Disease Registry (ATSDR) has a congressional mandate to assess the impacts of exposures to substances frequently found in the environment and to expand the knowledge base of health effects from exposure to hazardous substances [1]. To assist with the agency’s mission, a joint ATSDR and National Center for Environmental Health (NCEH) Microplastics Working Group (MPWG) was established. The MPWG has published four reviews: Microplastics Scoping Review of Environmental and Human Exposure Data, A Review of Data for Quantifying Human Exposures to Micro and Nanoplastics and Potential Health Risks, Worker Studies Suggest Unique Liver Carcinogenicity Potential of Polyvinyl Chloride Microplastics, and Systematic Review of Microplastics and Nanoplastics in Indoor and Outdoor Air: Identifying a Framework and Data Needs for Quantifying Human Inhalation Exposures. The effort in creating these reviews is time consuming, and the background and organizational work is traditionally made available only to the authors or their associates. While the full reference lists can be shared as supporting data with the journal and through scientific social media platforms, the organization of the data from the published findings performed for the reviews are not intuitive. Thus, the free sharing of full data elements is essential to increase the efficiency of reviews. To mitigate this issue and provide further advantages over traditional review formats, Hubs for Interactive Literature (HILs) were developed. HILs encompass all of the literature used in a review. Additionally, the references are categorized, can be filtered in an interactive manner, and include links or DOIs to the original papers. The ease of use enabled by the filtering options is difficult to overstate. The HILs reflect the tables and figures in the corresponding review in as true a manner as possible while also directly linking them to the relevant literature. A unique feature incorporated into each HIL is a searchable world map of references. These world maps quickly demonstrate where the research is taking place by displaying the number of references originating in each country in color gradient style. These maps allow fellow researchers to quickly find one another and hopefully establish new collaborations. Another example of an innovative use of the searchable world map is to identify the country of origin of specific types of research. In the case of microplastics, ATSDR was requested to help develop several expert panels that consisted of scientists in the economies that border the Pacific Ocean [2]. The expert panels consisted of no more than two experts from any one country. The world map feature will help to easily identify needed researchers in similar situations and thus foster global collaborations.

## 2. Data Preparation in Excel and Tableau Software

### 2.1. Data Preparation Excel^™^

The HILs described in the following sections were created using the Tableau software, but the first step for building a visualization is to create an Excel^™^ (Excel version 2308, Microsoft, Redmund, WA, USA) workbook with the desired data. There are many specific ways that Tableau^™^ (Tableau public software 2023.3.5, Tableau, Seattle, WA, USA) interprets data that must be addressed in the imported Excel workbook. Having a clear idea of how the data need to interact in the final visualization is essential to designing the data tables. Tableau^™^ allows for data in multiple spreadsheets to be joined to create tables, but the spreadsheets need to be related to at least one shared data field. For example, the interactivity for tables in the HILs is based on the unique IDs assigned to the references. This allows for Tableau^™^ to make connections between separate worksheets and even separate workbooks. The hierarchy of the data established in Excel is key to the look of the final tables. If the table being created has large categories that are further subdivided, then the imported data need to reflect this.

Our team first gathered all the literature used in the review and assigned the paper an ID number for organization purposes. That ID number comprised the first column. The second column was populated with the authors of the paper and the third column with the year of publication. The fourth column displayed the titles for each paper and the fifth column detailed the journal of publication. The sixth column was a hyperlink to the DOI or a URL to the publication and the seventh column was populated with the published abstract of the paper. In this way, any one ID was linked to the author, year, title, journal, DOI, and abstract for each of the papers in the review. In a separate Excel worksheet, for the creation of the world maps, we would again post the paper IDs as the first column; in the second column, we placed the DOI hyperlinks to the papers, and finally, in the third column, we placed the country where the paper was published out of. In this way, all IDs for the world map creation were linked to a DOI and country. We then would upload these worksheets into Tableau^™^ and begin the visualization process.

Sometimes, to achieve the desired design aspects, certain elements need to be repeated in multiple rows so that Tableau^™^ will read the data as pertaining to a single category. A beneficial feature is that not all the data in an Excel table need to be used in a table or graphic in Tableau^™^. Fields from multiple tables can be selected, and unnecessary data can be excluded. This allows for more flexibility compared to a typical table created in Excel.

### 2.2. Tableau^™^ Software

The microplastic (MP) HILs were created using Tableau^™^, an analytics platform focused on visual data analysis. Tableau^™^ enables the importation of data via spreadsheets, which can then be used to produce a large number of interactive graphics and tables. The software is available on both a free (public) platform and a version requiring a subscription. The version of the microplastic scoping review HIL described in Section 4 was produced collaboratively by SRC, Inc. and ATSDR in Tableau^™^ Public based on the scoping review by the ATSDR/NCEH MPWG. The additional HILs described in Section 5 are the original work of the MPWG.

## 3. HIL Content and Instructions

There are currently four HILs, which are complimentary to published reviews from the microplastic working group. All of the HILs begin with a couple of common features: a contents page and instructions pages. The contents page provides brief descriptions of the tables and figures in the visualization, as well as the categories that can be used in filtering them. Each HIL is designed to be as faithful to the reviews as possible and thus, the figures, table numbering, and content are maintained. The only area where this is not true is the interactive world maps, as those features are unique to the HILs.

The aspects explained in the instructions pages are hovering actions, accessing the DOI/URL of a reference, resetting the visualization, and filtering. The instructions are accompanied with images of the actions to enhance the user experience. Hovering over particular cells in a table or points in a figure will cause a ‘Tool Tip’ to display, which provides additional information. Cells where the complete information is not shown due to size restrictions are indicated with ellipses. Hovering over cells with ellipses will display all of the contents. This is a beneficial feature enabled by the HIL format, which prevents the tables from becoming too unwieldy and hard for the user to take in the data in an understandable manner. Many of the tables have small blue boxes at the end of the rows. Hovering over these boxes will display all of the information found in the row as a floating display, providing a succinct view of the data (Figure 1). In the world maps, hovering over a country will show the number of references from that country broken down by category, if applicable. Instructions for accessing the DOI/URL of the references directly from the visualization are provided. This is one of the most powerful features in the HILs. Finding the original reference(s) is simple, and the information is already organized by the topics and categorization of the review. This streamlines use of the information and references provided which is often difficult to accomplish in the standard review format. Brief descriptions for resetting visualization as well as filtering are also provided in the instructions. Filtering actions are explained in greater detail in the following section.

## 4. Example HIL Navigation

This section will provide an in-depth look at the HIL corresponding to the microplastics scoping review to demonstrate the various functionalities available. The purpose of the scoping review was to categorize the literature and better understand data gaps that are critical for future research. The process resulted in the inclusion of more than 500 articles that were separated into three broad categories: Absorption and Adsorption, Environmental, and Human Exposure [3,4]. The Absorption and Adsorption category focused on the chemistry surrounding microplastics and their interactions with external chemicals and microorganisms. The Environmental category concentrated on the presence of microplastics in the environment, further sorted by media including air, soil, sediment, and water. Finally, the category of Human Exposure comprised any literature that described human exposure to microplastics.

The scoping review identified 555 articles but only cited a small percentage (~10%) of them in the body of the work, making it difficult for researchers to identify and utilize specific literature. A Hub for Interactive Literature (HIL) was created as a complementary tool to add increased utility for the public and allow for maximal use of all the literature used in the review. The HIL employs a user-friendly and interactive format. The landing page of the HIL is displayed in Figure 1. The HIL comprises four sections: the categories and questions table (Figure 2a), the reference table (Figure 2b), the sources table (Figure 2c), and the abstract display (Figure 2d). The categories and questions table displays the numerical totals of all the literature used in the scoping review, broken down by both the broad categories and the 12 focusing questions in a heat map format that easily shows areas of both numerous and paltry research efforts (Figure 2a). These queries helped to focus the literature to identify essential data gaps, especially those vital to understanding and calculating human exposures and toxicity. For example, the categories and questions table clearly shows that 126, 419, and 10 papers in the Absorption and Adsorption, Environmental, and Human Exposure categories, respectively, are focused on microplastics, as addressed by Question 1. The reference table shows the ID number (assigned by the scoping review authors), authors, year published, title, and DOI/URL hyperlink for the papers. Hovering over the small square box at the far right of a row in the reference table displays a pop up with the complete abstract, author list, DOI, ID, source (i.e., journal the article is published in), title, type (journal article, book, or other), and the DOI hyperlink (Figure 1). By selecting the box, the full abstract for the paper will be shown in the abstract display (Figure 2d). The source table (Figure 2c) displays the number and percentage of the literature in each category as well as the classification by the source which includes journal article, review, and other (e.g., conference proceeding).

There are multiple ways to select areas of interest on the HIL, which filters the literature and changes the display; a few of those methods are described here. In the categories and question table, selecting one of the three categories at the top right will cause the references table to display only the literature in the chosen category. The category can also be selected in the left column of the source table. Additionally, the functionality of the source table allows the user to further filter the literature by selecting any of the cells. For example, selecting the first cell in the journal article column will cause both the reference table and the category and questions table to only display journal articles in the Absorption and Adsorption category (Figure 3). Forward, backward, and reset options are available at the bottom of the HIL, enabling further user capabilities. Overall, the scoping review HIL allows users to view and, most importantly, rapidly filter hundreds of sources focusing on microplastics into niche areas. We believe that the ease of use this tool offers will increase efficiency by removing some of the time intensive barriers involved with the stand-alone traditional review format.

An innovative feature added to the scoping review HIL, and every subsequent HIL, is the searchable world map(s) (Figure 4). This feature is organized into four distinct sections: the references table, the category table, the maps, and the search bar. The references table is divided into seven categories: Country, Category, ID, Author, Year, Title, and DOI. If the user selects a specific country in the references table, that selection will influence both the category table and the maps. The Category column refers to the three broad categories in the scoping review (Environmental, Absorption and Adsorption, and Human Exposure). The ID column displays the papers’ ID, which is consistent throughout the HIL. The DOI column displays the DOI hyperlink, which, when clicked, will take the user directly to the official publication.

The category table is a color-coded, heat-map-style table that displays the three general categories used in the scoping review across the top on the horizontal axis and the countries along the vertical axis. Each cell is populated with the number of publications in that category originating from a specific country. The color scale of the cells ranges from white to dark blue, which correspond to a count of 0 and 81, respectively. From this table, the user can easily see the number of publications, by category, produced by each country. The category table is interactive, and any selection will influence both the reference table and the maps.

For this HIL, there are three interactive maps representing the three categories from the scoping review. The maps use colors in two ways. The first is simple differentiation, where the Adsorption and Absorption map is orange, the Environmental map is green, and the Human Health map is purple. Second, color is also used as a gradient where the hue of color reflects the number of publications. The darker the color a country is, the more publications of that category there are. The maps are interactive; when the user selects a country on the map, the reference table and category table in turn will highlight the chosen country and display the information associated with it.

Finally, there is a search function located over the references table. This tool allows users to search through this visualization by year, category, and country. The user can type in a year, such as, “2020”, and this will update the reference table with all publications specifically published in the year 2020. Searching one of the three categories will update both the reference table and the category table with publications pertaining to only the chosen category. Finally, users can search by country, and this will adjust both the reference table and the category table to only data pertaining to the specified country. For example, if a user wanted to determine how many publications focused on the environmental category from China, the user could type in ‘China’ into the search tool above the reference table (Figure 4). This would populate the reference table strictly with all publications authored in China. This would also populate the category table with the country China, showing the number of papers by category. The user could select the desired category and be able to view all of the relevant publications from China. From the reference table, users can also directly interact with the DOI column to go to the selected papers’ publication page.

## 5. Other HILs

We will briefly describe the three other HILs in this section and outline their unique features. Each visualization has multiple tabs, the first tab contains the contents of the visualization. The second and third tab contain instructions for use. All subsequent tabs are comprised of figures and tables that are as faithful to the original papers as possible. As HILs are intended to be complementary visualizations the labeling is consistent with the papers. Thus, they are named, “Table 1, Table 2, Table 3, Table 4, etc.” in accordance with the papers that the HILs are based on.

The MPWG’s second review focused on microplastics that have been measured in air, soil, foods, and beverages. This publication also included estimates of exposure and health effects [5]. The corresponding HIL contains seven interactive tabs reflecting the figures from the original publication. Generally, each table or figure in the review is translated into one tab of the HIL visualization. With Tables 1, 2, and 3 (each represented by a separate tab in the visualization) users can interact with the category bar at the top of the pages to narrow down publications by category. For example, if the user selects “Seasonings” in the category bar of Table 1, this will update the data table (directly below category table) and references table (the bottom most table) to show only data and bibliographic information pertaining to microplastics in seasonings, respectively (Figure 5). Table 2 functions similarly to Table 1, but the categories instead focus on the type of microplastic: PVC, flock, or textile. Table 3 has a similar format to Tables 1 and 2 in terms of the category bar being used as a filtering feature. Table 3 provides the data on microplastics exposure, markers, and health effects in both animals and humans. It is organized by the following categories: biological response, detection of general exposure, food and GI, and surgical implant.

Table 4 from the human exposures review is divided into three separate tabs. Similar to the previous tables, filtering interactivity is possible with the category bar at the top of the visualization. The three tabs comprising Table 4 have unique features not seen in the previous three (Figure 6). The cells in the tables are each color-coded either green, yellow, or red. Green reflects sufficient characterization to demonstrate the presence of microplastics, yellow shows the need for further characterization, and red indicates cells with the largest data needs. All of the Table 4 tabs also have independent sections describing microplastics and nanoplastics. Finally, this HIL has a world map tab that functions in the same manner described in the previous section.

### 5.1. Potential Liver Carcinogenicity of PVC Microplastics-HIL

This HIL enables a focused screening of the data to identify only those studies associated with a particular microplastic substance, which reflects the related review. This feature is helpful in comparing health effects due to a single microplastic exposure, such as to polyvinyl chloride (PVC), to many other specific microplastic substances [6]. The HIL contains 3 interactive tabs reflecting the figure from the original publication. The publication contains one large table that displays the type of microplastic as the main category and then shows the various studies reflecting that microplastic type and factory worker exposures to those microplastics. It should be noted that in the review, this table is over five pages long. However, in the HIL, this translates to a single page including filtering options. This again demonstrates the advantages the HIL tool possesses in terms of usability. The Potential Liver Carcinogenicity of PVC Microplasitcs-HIL Table 1 tab has an interactive category row at the top of the visualization where the user can select the type of microplastic. Selecting the type of microplastic will populate two lower tables, the data table and the reference table. The data table (the first lower table) will show a brief description of the pertinent exposure information from the various publications that showed exposure with the specific microplastic type chosen. This information is displayed as the industry, study details (such as cohort size), hepatic effects, respiratory and cardiovascular effects, gastro/colon effects, and other effects. The reference table (lowest table) will populate with related publication data, such as author, title, abstract, and DOI, to allow the user to easily find the original publication. This particular HIL has two separate map tabs. The first map tab specifically represents all the publications and data can be found in Table 1. The second map is the traditional map included in all the HILs, reflecting all the literature cited in the review.

### 5.2. Review of MPs and NPs in Indoor and Outdoor Air HIL

The companion HIL to the MPWG’s recent review on microplastics and nanoplastics (NPs) in indoor and outdoor air offers several unique features based on the original figures and tables. Researchers interested in air concentrations and exposure can also quickly filter those published works used to identify the types of microplastics found in air and concentrations collected in indoor or outdoor air by geographic region and develop a framework to calculate air exposures [7]. In this work, 4863 articles were available for screening, which were distilled to 258 articles for referencing, and used to derive exposure estimates. The paper contains a flow chart of the literature focused on exposure assessment and health outcomes that is translated into an interactive table in the HIL. A useful feature that is enabled by the HIL format is the reference display that is linked to the table. Instead of merely seeing a number and a list of first authors, selecting a category in the table filters the reference table to display only the related references with quick access to the DOI links.

Microplastics in air review has some data tables with corresponding bar charts to provide both the exact numbers for the exposures in various locations and age groups and an easy visual for comparisons. In the companion HIL, a data table and its related chart are presented on the same page along with a references table. The advantages of the tool tip are again demonstrated in this format. Hovering over a bar in the chart will provide the age group, the sampling location, and the dosage, which is the information provided by the data table. This quickly provides the user with the combined chart and table information, which is not possible in a published review. The visualization also offers helpful filtering options. Selecting a bar in the chart will adjust the data table to only display the information on the specified location and cause the reference table to show the related literature (Figure 7). On both the data table and the bar chart, selecting an age group adjusts the rest of the page to reflect only the relevant data. Additionally, the data tables are shown in an easily interpretable heat map format, which adds an extra element to the table presentation compared to the original review. The searchable world map in this particular visualization is especially powerful as it shows the countries of origin for where the sampling took place. As micro and nanoplastic conditions vary widely depending on the type of environment and location, this adds a further dimension to the available data that is not possible in the review.

## 6. Conclusions and Potential Advantages of HIL Utilization in Future Reviews

Hubs for Interactive Literature (HILs) allow users to easily manipulate all the literature used in the related reviews, enabling them to better understand the results of the publications. This manipulation will also allow users to more readily identify potential new relationships within the review literature, answer specific questions regarding the data, and realize relationships/associations between data beyond what is specifically in the review itself. We have added, and plan to add, to every future HIL a tab specifically mapping the geographic locations of where the studies were conducted. HILs, particularly the map features, will help connect scientists and the public alike to further research on microplastics.

The pairing of interactive HILs with reviews is the next big leap in the state of review science. Scientific reviews compile large amounts of primary literature and synthesize that literature into useful data. However, this often results in difficulty locating the primary sources for the reader. Searching through the primary literature can often be a time consuming and stifling process. Instead, presentation of the references in an interactive database format allows researchers to rapidly find the papers they need, ultimately reducing the time required to complete experiments inspired by or based on existing literature reviews. We plan to post and release the HILs later this year.

## Figures and Tables

**Figure 1.**
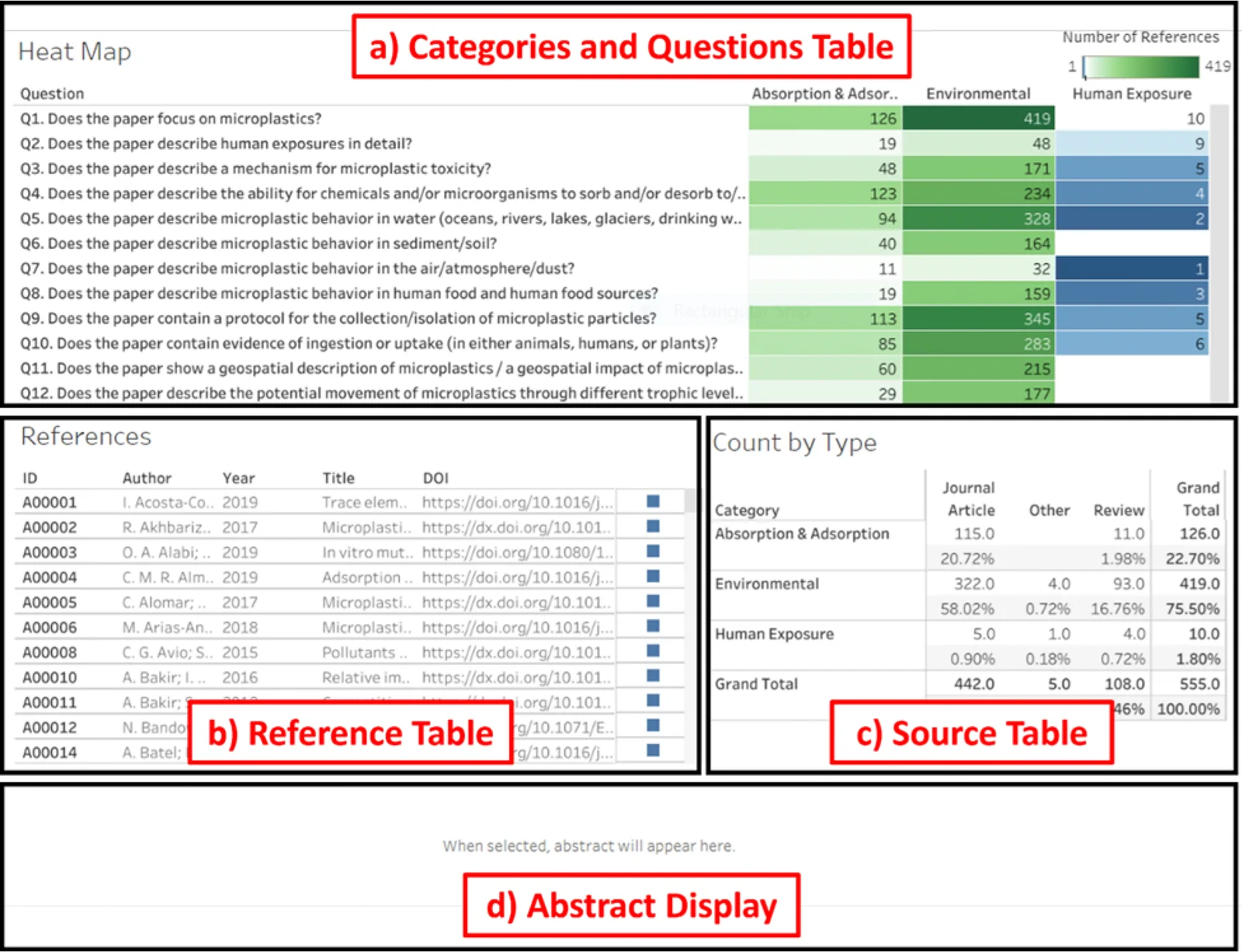
Example Individual Reference Display Window. When an individual reference is selected by clicking on the small square box at the end of a table row, additional information including the abstract is displayed.

**Figure 2.**
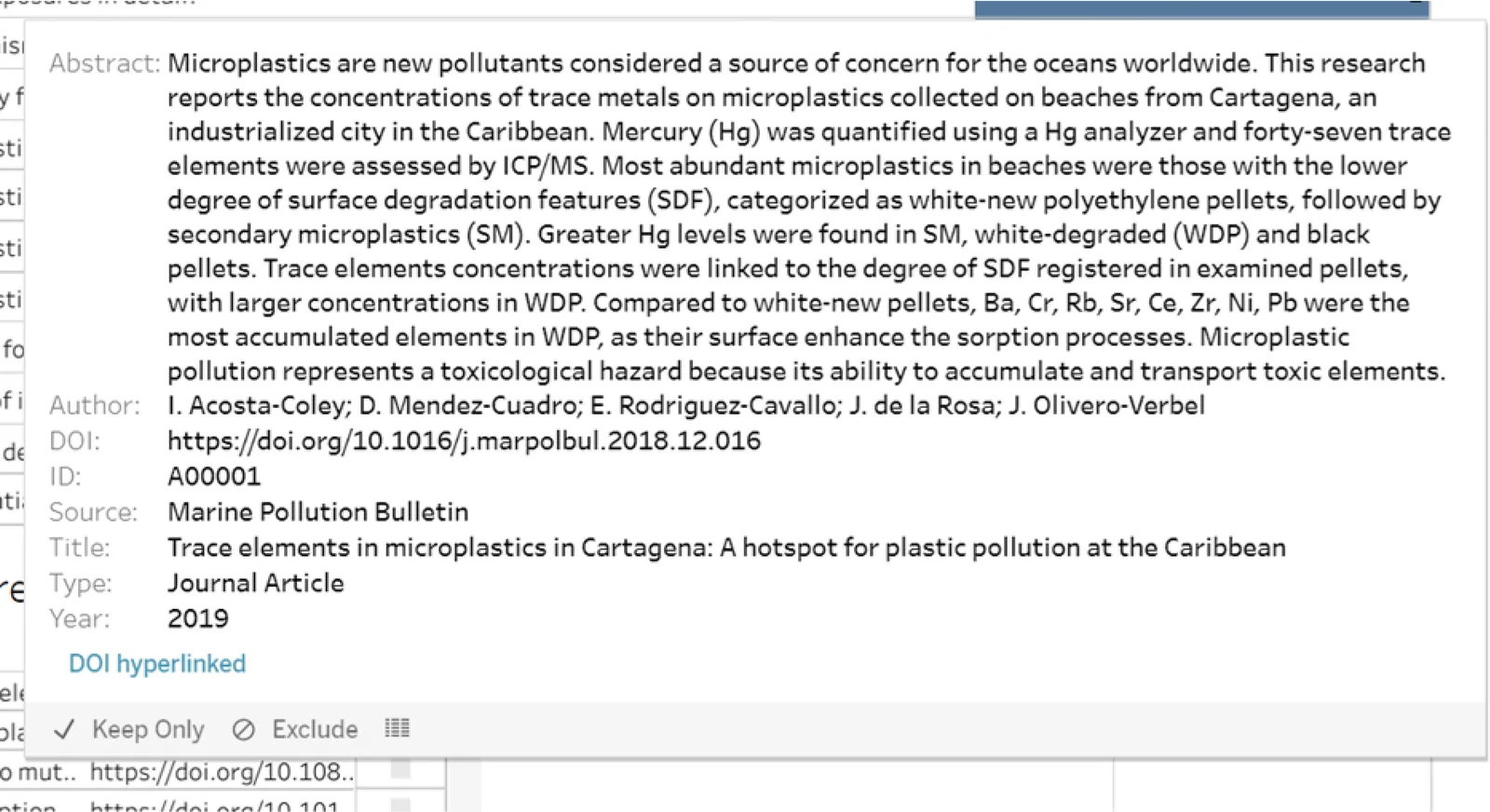
The landing page of the microplastics scoping review HIL. The sections of the HIL are (**a**) the categories and questions table, (**b**) the reference table, (**c**) the sources table, and (**d**) the abstract display. Please note that the alphabetic labels are added to allow for ease of description and do not appear on the live HIL.

**Figure 3.**
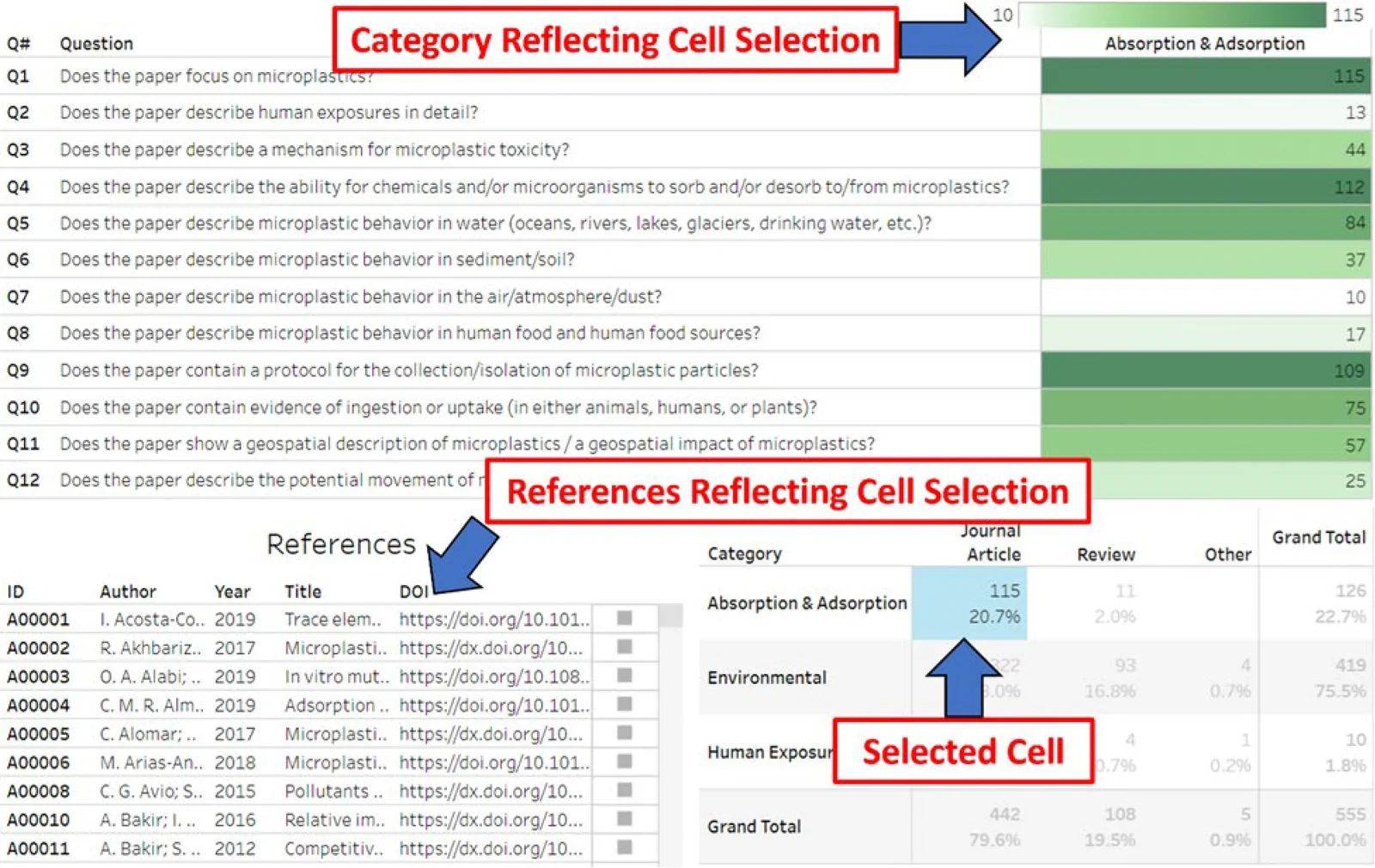
An example of a result from a scoping review HIL filtering option. The results shown reflect selecting only the journal articles in the absorption and adsorption category using the sources table.

**Figure 4.**
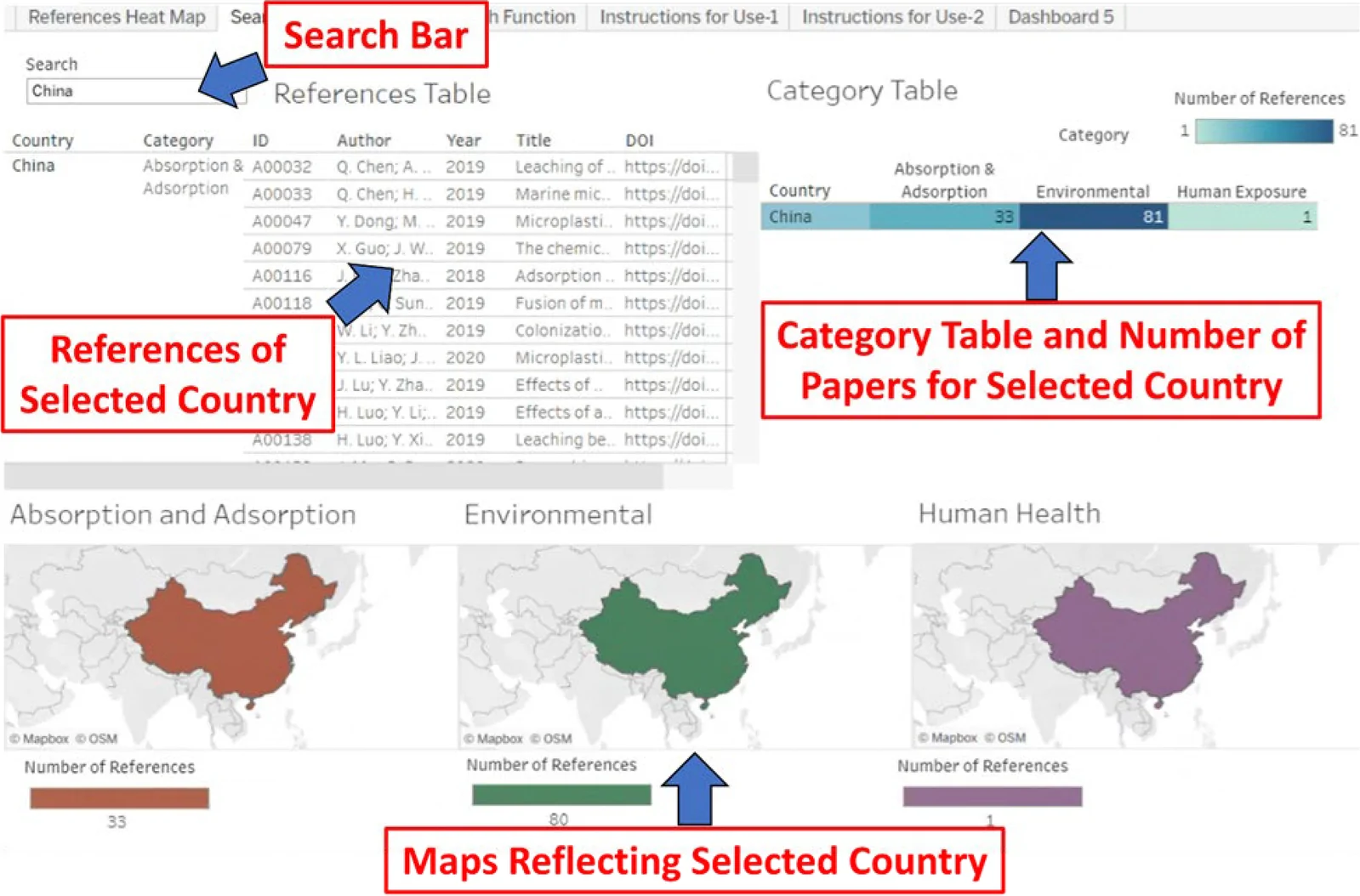
An example of the search bar function for the world map. The key features of the world map page, the search bar, reference table, category table, and interactive maps, are indicated. The results of searching for ‘China’ in the search bar are shown.

**Figure 5.**
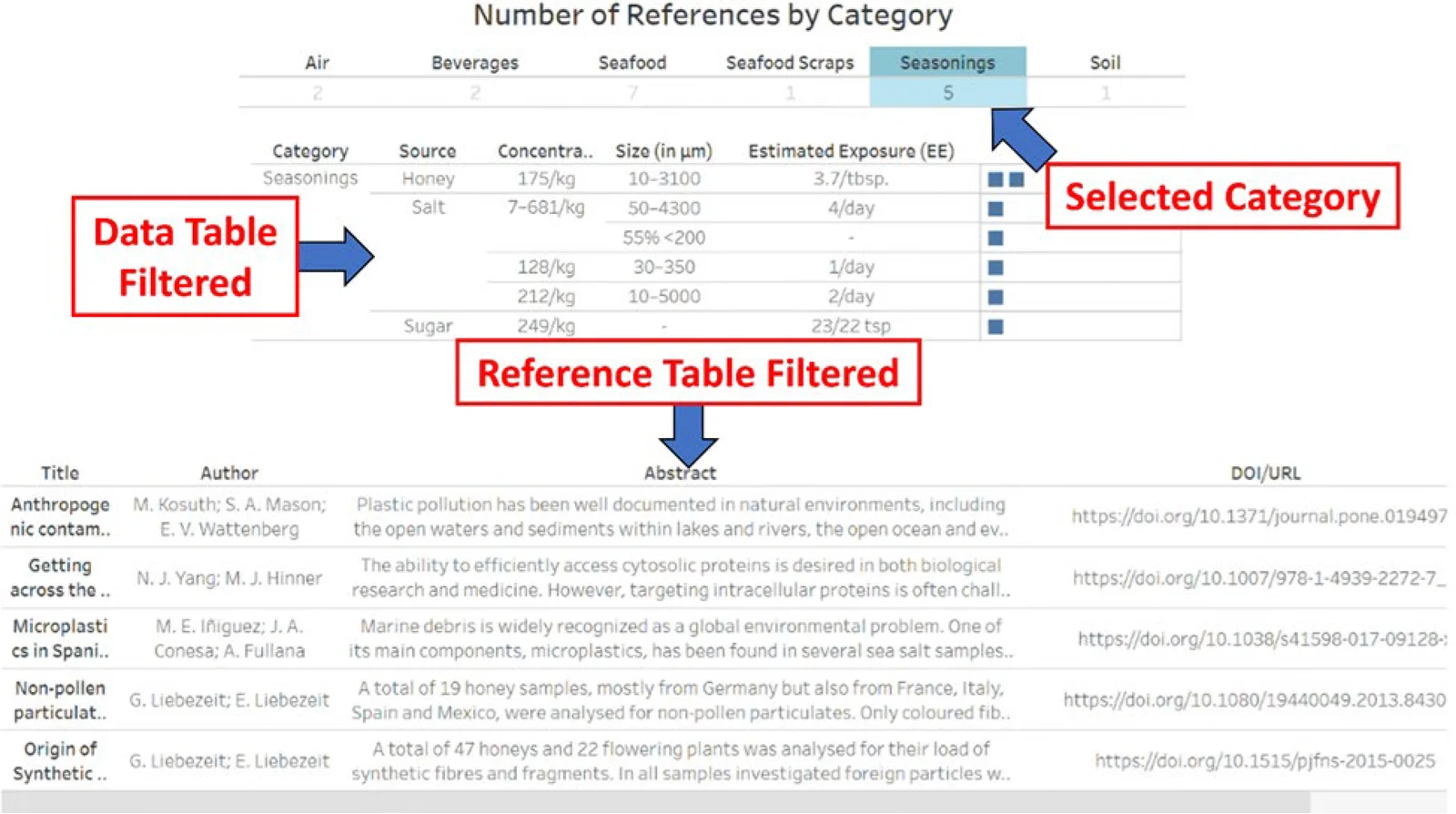
Table 1 tab reflecting the selection from the category bar. ‘Seasonings’ is selected in the category bar. The selection results in the filtering of the data table and the reference table.

**Figure 6.**
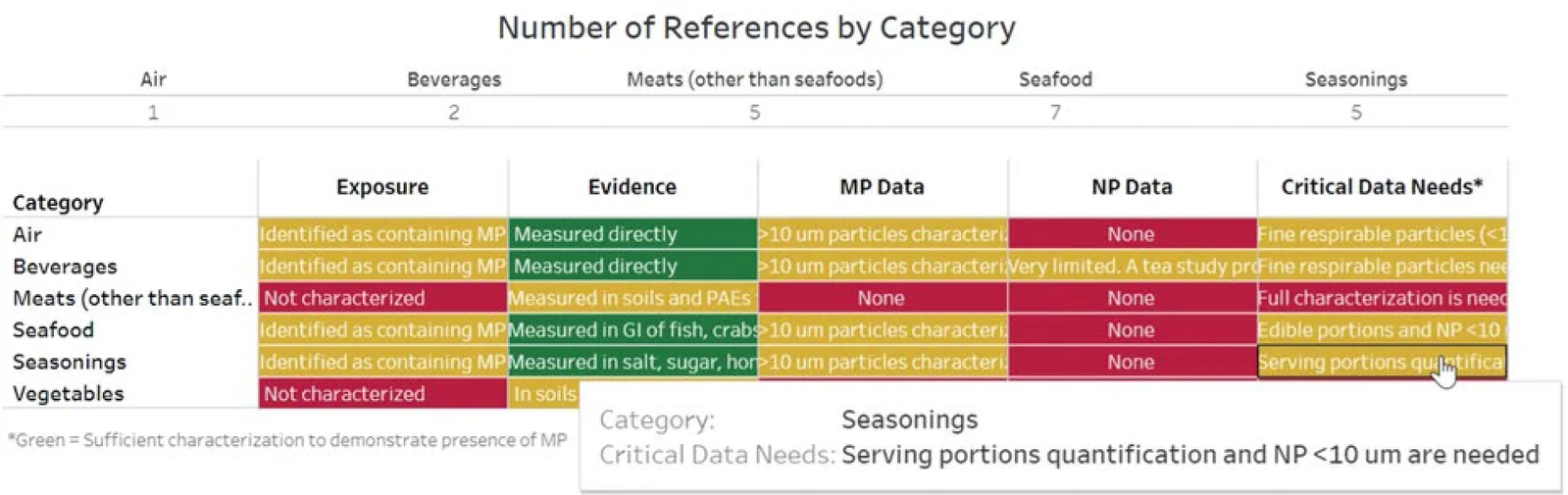
Table 4–1 tab demonstrating the hovering over ellipses. This table is color-coded to replicate that in the original review. The advantages in terms of space and readability of the hovering option are demonstrated.

**Figure 7.**
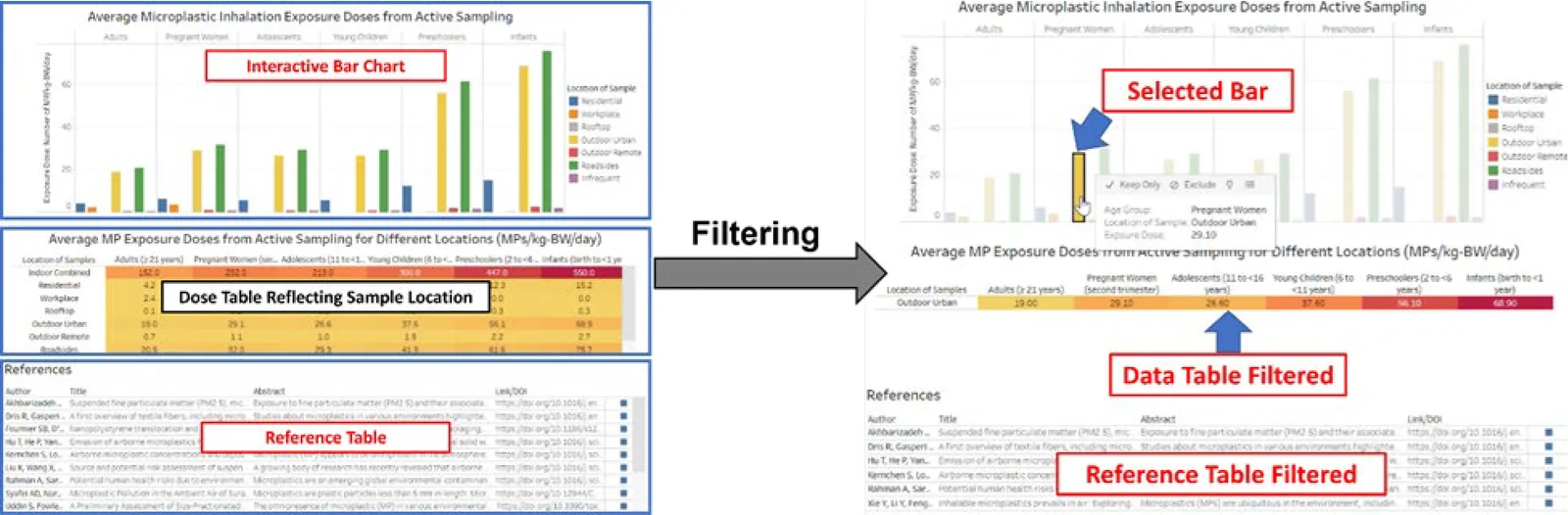
The table 1 and figure 4 tab in the Review of MPs and NPs in indoor and outdoor air HIL visualization shows an example of filtering one of the interactive bar chart and data table pairs. The information provided by the tool tip is also shown.

## Data Availability

Data are contained within the article.
